# DNA methylation dynamics during embryonic development and postnatal maturation of the mouse auditory sensory epithelium

**DOI:** 10.1038/s41598-018-35587-x

**Published:** 2018-11-26

**Authors:** Ofer Yizhar-Barnea, Cristina Valensisi, Naresh Doni Jayavelu, Kamal Kishore, Colin Andrus, Tal Koffler-Brill, Kathy Ushakov, Kobi Perl, Yael Noy, Yoni Bhonker, Mattia Pelizzola, R. David Hawkins, Karen B. Avraham

**Affiliations:** 1 Department of Human Molecular Genetics and Biochemistry, Sackler Faculty of Medicine and Sagol School of Neuroscience, Tel Aviv University, Tel Aviv, 6997801 Israel; 2 Division of Medical Genetics, Department of Medicine, Department of Genome Sciences, Institute for Stem Cell and Regenerative Medicine, University of Washington School of Medicine, Seattle, WA 98195 USA; 3 Center for Genomic Science of IIT@SEMM, Fondazione Istituto Italiano di Tecnologia, Milano, 20139 Italy

## Abstract

The inner ear is a complex structure responsible for hearing and balance, and organ pathology is associated with deafness and balance disorders. To evaluate the role of epigenomic dynamics, we performed whole genome bisulfite sequencing at key time points during the development and maturation of the mouse inner ear sensory epithelium (SE). Our single-nucleotide resolution maps revealed variations in both general characteristics and dynamics of DNA methylation over time. This allowed us to predict the location of non-coding regulatory regions and to identify several novel candidate regulatory factors, such as Bach2, that connect stage-specific regulatory elements to molecular features that drive the development and maturation of the SE. Constructing *in silico* regulatory networks around sites of differential methylation enabled us to link key inner ear regulators, such as Atoh1 and Stat3, to pathways responsible for cell lineage determination and maturation, such as the Notch pathway. We also discovered that a putative enhancer, defined as a low methylated region (LMR), can upregulate the *GJB6* gene and a neighboring non-coding RNA. The study of inner ear SE methylomes revealed novel regulatory regions in the hearing organ, which may improve diagnostic capabilities, and has the potential to guide the development of therapeutics for hearing loss by providing multiple intervention points for manipulation of the auditory system.

## Introduction

The mammalian inner ear is a highly complex organ that is responsible for hearing and balance^1,2^. Both functions are crucial for the survival and development of the organism throughout its lifetime. Defects in either the structure or function of any part of the inner ear sensory organs may result in auditory and vestibular impairments and are responsible for the most diverse variety of genetic disorders^3–5^. The sensory organ responsible for hearing, the cochlea, contains the sensory epithelium (SE)^1,2^. This region is composed of sensory cell types, i.e., the hair cells, as well as non-sensory cell types, i.e., the supporting cells. Transcriptomic approaches have provided information about the genetic determinants that drive each decision during the development and differentiation process of the inner ear sensory organs^6–8^. RNA high-throughput sequencing studies of the SE have led to the identification and characterization of non-coding RNAs (ncRNA), including microRNAs (miRNAs) and long non-coding RNAs (lncRNAs)^9,10^. RNA transcriptomic analysis at the single-cell level has been undertaken only recently in this tissue^11–13^, setting the stage for understanding the role of the individual cell types encompassing the SE tissue. However, to date, epigenetic analysis of the SE is still lacking, due to the technical challenges involved in the separation of cell types, and the low yield of material available for subsequent analysis. In recent years, technological advances in the epigenomics field have opened the door to unprecedented opportunities for unraveling of the epigenetic regulation of SE development and maturation. Transcriptomic and epigenomic analyses of the auditory system will lead to a better understanding of the genetic program of SE development and maturation and may hold the key to enable genetic manipulation and regenerative medicine in inner ear-related pathologies, including deafness.

DNA methylation remodeling is an essential component of epigenetic regulation during development^14^. This study was designed to elucidate DNA methylation dynamics during mouse SE development and maturation. To this end, we generated single nucleotide resolution genome-wide maps at key developmental time points and built a regulatory network underpinning tissue transitions throughout the developmental process that culminates in a functional, hearing inner ear. We discovered a close association between methylation dynamics through key developmental time points and transitions of the mammalian inner ear, with implications for the regulation of major signaling pathways such as Wnt and Notch, and mechanisms such as neurogenesis. In addition, the analysis provides the basis for an expanded view of the regulatory mechanism of G*JB6*, a critical gene in human deafness. This study is the first to report a single-base resolution methylome of the mammalian inner ear and provides information about regulatory pathways defining sensorineural hearing loss and deafness. The results obtained from exploiting this unique resource shed new light on the complexity of developmental and pathological mechanisms of both hearing and deafness in humans.

## Results

### Inner ear sensory epithelium methylomes

**Figure 1:**
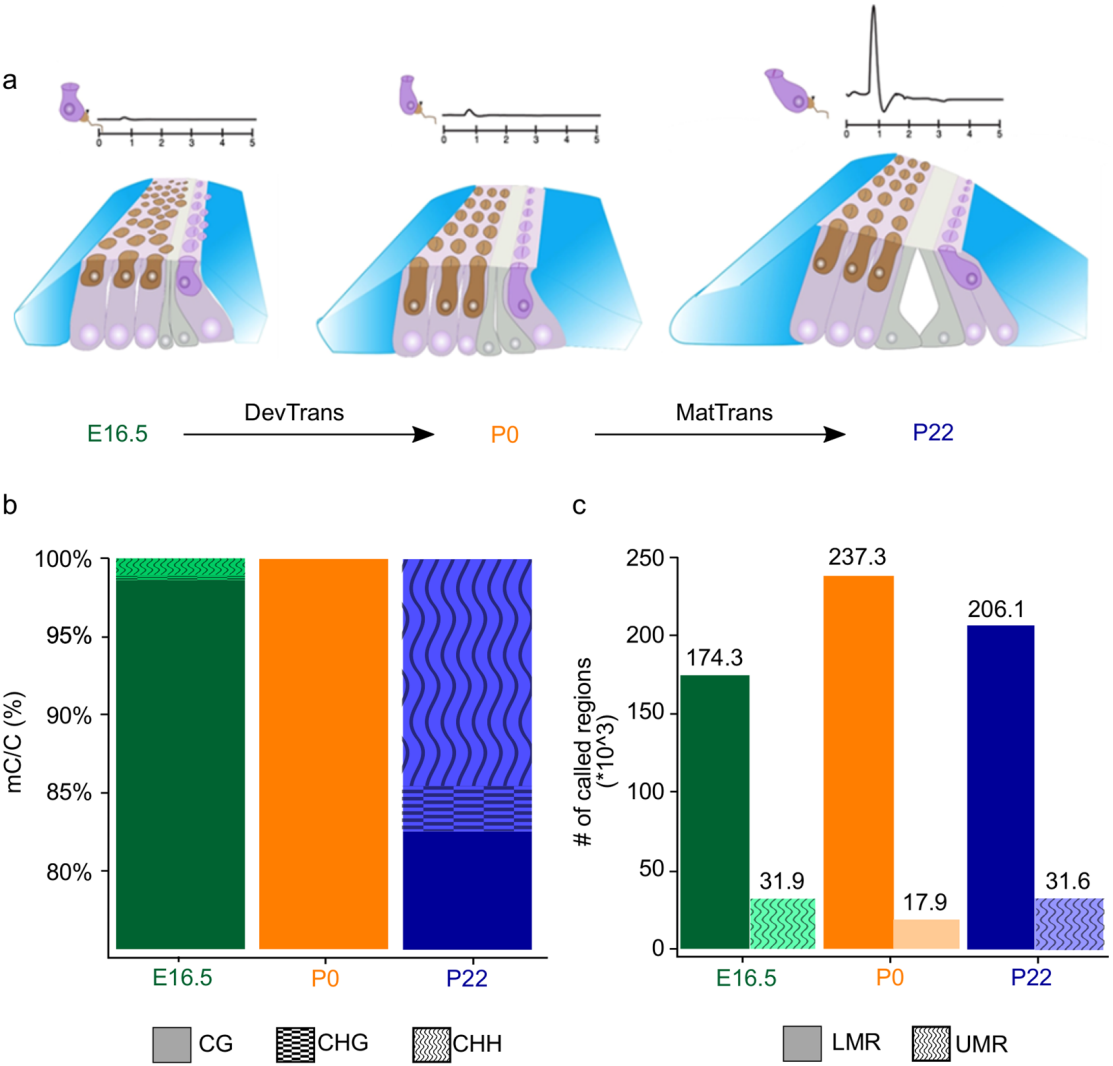
General features of inner ear sensory epithelium (SE) methylomes. (**a**) Illustration of the inner ear SE composition of sensory hair cells (brown, dark purple) and non-sensory supporting cells (blue, grey, light purple) at E16.5, P0, and P22. Representative auditory brainstem responses (ABR) are shown above each time point. DevTrans: Development Transition, represents the methylation dynamics for the P0 compared to E16.5; MatTrans: Maturation Transition, represents the methylation dynamics for the P22 vs P0 development. (**b**) Distribution of all methylated cytosines in CpG (mCG) (solid), non-CpG CHG (checkered), and non-CpG CHH (waved) contexts for the three time points. The figure represents merged data from two independent biological replicates for each time point. The average genomic coverage was 19.5X in E16.5, 11.1X in P0, and 15.1X in P22. (**c**) Number of low-methylated regions (LMRs) (solid) and unmethylated regions (UMRs) (dotted) at each time point.

Studies in tissues monitoring changes in DNA methylation patterns have revealed critical information about development and gene regulation^15–17^. The resolution of sequencing-based approaches permits the detection of tissue-specific non-CpG methylation, differential methylation, and of *cis*-regulatory elements, and has revealed correlations with changes in expression. To explore how DNA methylation dynamics may contribute to regulate inner ear development and maturation, we generated single-base resolution whole genome methylomes of the mouse inner ear SE for three key developmental stages: embryonic day 16.5 (E16.5), postnatal day 0 (P0), and postnatal day 22 (P22) (Fig. 1a, Supplementary Fig. S1a). At P0, prosensory and non-prosensory cell specification has already occurred and the SE is in the process of developing into a single row of sensory inner hair cells and three rows of sensory outer hair cells, surrounded by non-sensory supporting cells. Although at P0 the cells are already post-mitotic and the tissue can be considered non-proliferative, the SE is not yet functional and the mice cannot hear. By P22, the SE is fully mature and mice have acquired hearing^18^.

DNA methylation predominantly occurs at cytosine and its function depends on the sequence context. While methylated cytosines are mostly located in the context of CpG dinucleotide (mCG), the methylation in non-CpG contexts (mCHH and mCHG, where H = A, T or C) has been shown to occur in some cases, such as adult mammalian brain^15,19^ and embryonic stem cells^20^. To assess DNA methylation in both the CG and non-CpG (CHG and CHH) contexts, we generated 1.3 billion unique mapped reads, combining two biological replicates per each time point and with an average genomic coverage ranging from 11X to 19.5X (Supplementary Fig. S1b). As expected, most of the methylated cytosines are in the mCG context (Fig. 1b). Interestingly, at P22, we observed a prevalence of mCH methylation in both CHG and CHH, the former representing 2.9% of all methylated cytosines, and the latter 14.6% (Fig. 1b). While CH methylation is present in both mouse and human embryonic stem (ES) cells, it is largely lost upon cell differentiation^21,22^. Recent evidence has shown that mCH methylation accumulates specifically in neurons during development, although the function is still unclear^15^. Thus, the increase in mCH we observed upon maturation of the SE might be associated with onset of function at both ends of the auditory system, the auditory cortex in the brain and the mechanosensory SE in the inner ear.

### Regulatory landscape of the inner ear SE

Previous studies revealed that *cis-*regulatory elements such as promoters and enhancers can be identified in whole genome bisulfite sequencing (WGBS) data through the detection of unmethylated and low-methylated regions (UMR/LMRs), respectively^23,24^. To identify putative regulatory elements, such as promoters and enhancers, in the SE, we defined UMRs as regions with an average methylation lower than 10%, and LMRs as regions with an average methylation between 10% and 50% (Supplementary Fig. S1c,d). With these conditions, we identified 31,916, 17,945 and 31,564 UMRs and 174,347, 237,282 and 206,144 LMRs in E16.5, P0, and P22 methylomes, respectively (Fig. 1c; Supplementary Table S1). UMRs were validated by their localization to annotated transcription start sites (TSS) and CpG islands (CGIs) (Supplementary Fig. S1e,g). In contrast, LMRs were predominantly located far from TSS in intergenic or intronic regions and were generally devoid of CGIs (Supplementary Fig. S1f,h).

**Figure 2:**
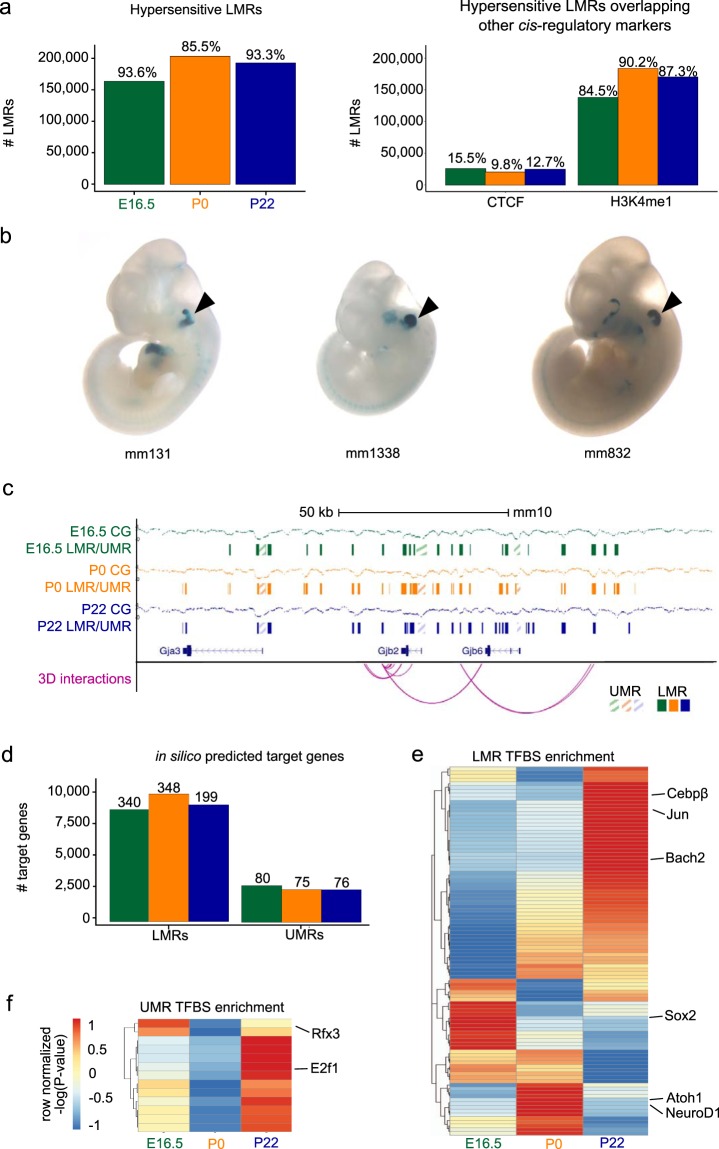
Putative regulatory landscape of the inner ear SE. (**a**) Right bar plot shows the number of low-methylated regions (LMRs) overlapping known DNase I Hypersensitive Sites (DHS) from the mouse ENCODE project^25^. The numbers on each bar represent the percent overlap with respect to all LMRs. Left bar plot shows the number of hypersensitive LMRs that overlap CTCF binding sites and H3K4me1 enhancer peaks. The numbers on each bar denote percent overlaps. (**b**) Examples of experimentally validated mouse non-coding fragments with otic (ear) enhancer activity as assessed in transgenic mice from Vista Enhancer Browser for which we found an overlap with LMRs at one of the three time points. (**c**) Browser shot of the *Gjb2* gene locus, illustrating percent methylation levels, LMRs, unmethylated regions (UMRs) and their putative interactions with target genes. (**d**) Bar plot showing the number of putative target genes interacting with LMRs and UMRs. The numbers on each bar denote the count of known deafness target genes. (**e**, **f**) Heatmap of row normalized -log(P-value) for the relative enrichment across the three time points for transcription factor (TF) motifs present in LMRs (**e**) UMRs (**f)** and filtered for expression. Representative TFs are shown on the side.

To ensure that LMRs were representative of known distal regulatory elements, we examined whether they overlapped known DNase I Hypersensitive Sites (DHS) in the mouse genome, which are indicative of transcription factor (TF) binding. To do so, we leveraged the power of a large consortium project, Encyclopedia of DNA Elements (ENCODE)^25^, where epigenomic data has been collected across numerous tissues, cell types, and cell lines. Using data from mouse ENCODE, we found that 93.6%, 85.5%, and 93.3% of LMRs from E16.5, P0, and P22, respectively, were DHS in other mouse tissues (Fig. 2a). Out of the LMRs that included a known DHS, 9.8–15.5% contained a CTCF motif and overlapped known CTCF binding sites^26,27^, suggesting a role as insulators or in three-dimensional (3D) genome architecture (Fig. 2a). The remaining 84.5–90.2% overlapped known H3K4me1 sites from the mouse ENCODE project (Fig. 2a)^26,27^, suggesting they may function as enhancer elements^28^.

Further evidence that LMRs can act as putative enhancers, comes from the observation that 80% (12 out of a total of 15) of functionally validated mouse otic enhancers from the VISTA Enhancer Browser, a resource for experimentally validated human and mouse non-coding fragments with gene enhancer activity assessed in transgenic mice, are recovered by our LMRs (Fig. 2b; Supplementary Table S2). Collectively, these findings show that identification of LMRs is a valuable approach to annotating putative regulatory elements in the mouse genome related to SE gene regulation. In order to understand the regulatory implications of these elements, we determined their target genes. Distal UMRs, such as those at non-promoter CGIs, and LMRs were putatively assigned to target genes. We used the 4DGenome database for known interactions and the PreSTIGE (Predicting Specific Tissue Interactions of Genes and Enhancers) database for predicted interactions, a computational approach for defining enhancer–gene interactions, which integrates available H3K4me1 ChIP-seq and RNA-seq datasets and couples cell type-specific H3K4me1 signals with genes expressed in each cell type^29^. Importantly, we were able to assign a number of distal UMRs and LMRs to functionally relevant genes with known roles in mouse inner ear development, and hearing impairment or deafness (Fig. 2c,d; Supplementary Table S3)^30^. For each developmental time point, an average of 77 and 296 genes could be linked to distal UMRs and LMRs, respectively.

In addition, the presence of significantly enriched transcription factor binding site (TFBS) motifs in UMRs and LMRs was used in order to predict putative regulators of target genes (Fig. 2e,f; Supplementary Table S4). TFBS were screened for TFs expressed at the various developmental time points according to available transcriptomes^8–10^. Our approach resulted in the identification of TFBS of several known inner ear SE master regulators (e.g. Atoh1, Sox2, NeuroD1, and Rfx3)^6,8^. We also identified E2f1, which is linked to maternally inherited deafness originating from aberrant methylation and an increase in this pro-apoptotic TF^31^. Interestingly, we found that a Bach2 motif was significantly enriched at LMRs across all time points. Bach2 is known for its role in the Bcl6-Bcl2-p53 axis-controlling apoptosis (reviewed in^32^), together with otic expression in the chick^33^, although it has not been previously reported to play a role in inner ear development.

### DNA methylation dynamics during development and maturation transitions

**Figure 3:**
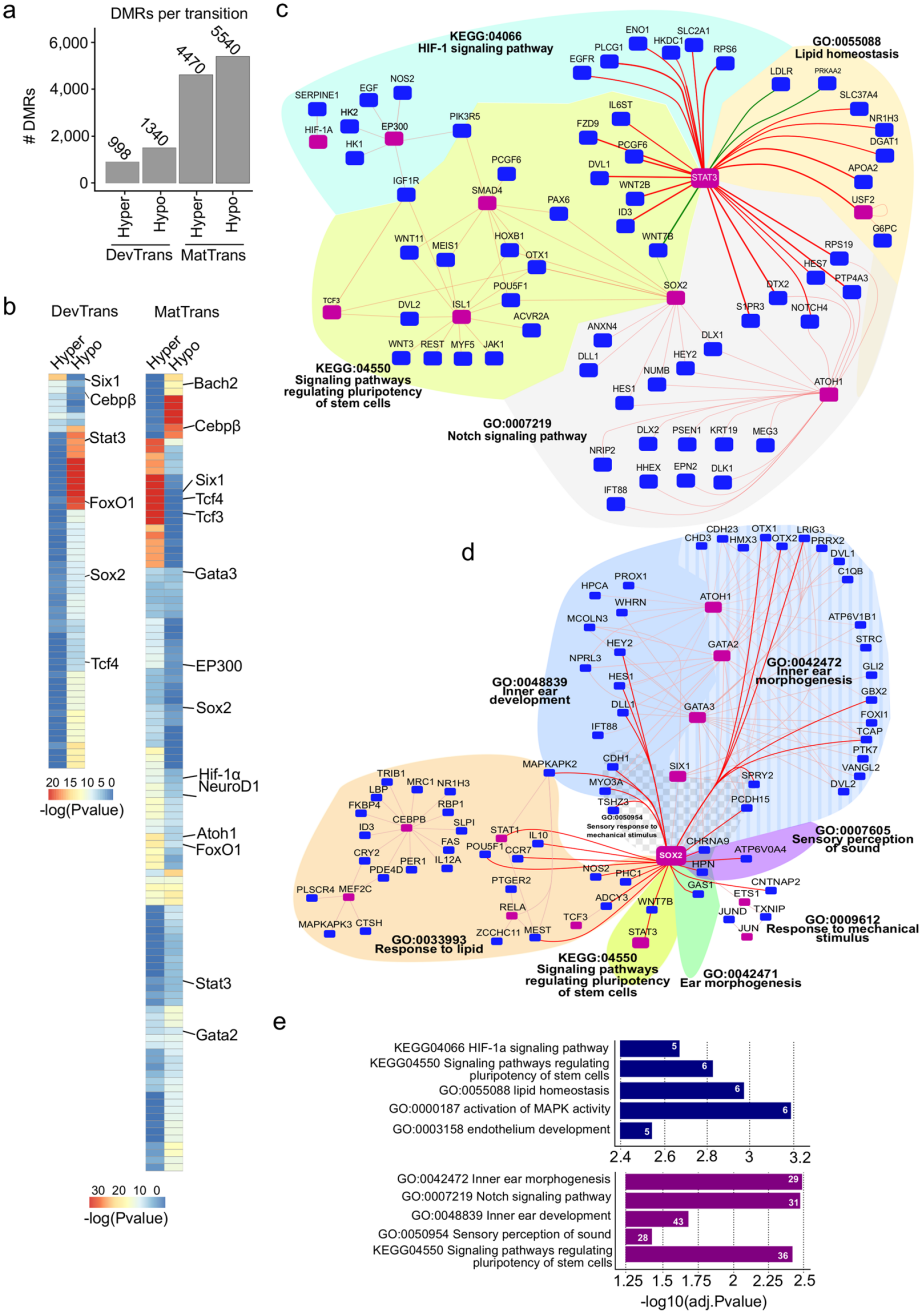
Methylation dynamics across development and maturation transitions. (**a**) Bar plot showing the number of hyper- and hypo-differentially methylated regions (DMRs) identified in DevTrans and MatTrans. (**b**) Heatmap representation of enriched transcription factor binding site (TFBS) motifs in DMRs for each transition. (**c**, **d**) *In silico* transcriptional regulatory networks based on DMR target gene interactions during DevTrans (green connecting line) and MatTrans (red connecting line). Known deafness transcription factors (TFs)/genes are marked by purple squares. TF-target gene interactions are clustered according to common GO terms (indicated by various color and patterned filled areas). Centralized specific TF *in silico* transcriptional regulatory networks around the hair cell marker, Stat3 (**c**) or supporting cell marker Sox2 (**d**). All direct interactions with centralized TFs are indicated by bold lines. (**e**) The enriched GO biological process terms and pathways for the overall regulatory networks of DevTrans (top) and MatTrans (bottom) (Table S7), number of GO term connected genes are shown in white.

To explore how DNA methylation dynamics may contribute to regulate inner ear SE development and onset of hearing, we focused on two comparisons: E16.5 to P0, which we refer to as the “developmental transition” (DevTrans), and P0 to P22, which we refer to as the “maturation transition” (MatTrans) (Fig. 1a). We then determined differentially methylated regions (DMRs), where there was at least a 30% loss (hypo-DMR) or gain (hyper-DMR) in methylation across the transition (P-value < 0.05; Fig. 3a; Supplementary Fig. S2a,b, and Table S5). Our results indicated higher dynamics in MatTrans (~10,000 DMRs) than in DevTrans (~2,300 DMRs), although the ratio of hypo- and hyper-DMRs was similar for each transition. Because DMRs are primarily located in intronic and intergenic regions, they overlap LMRs more frequently than UMRs (Supplementary Fig. S2c,d). Collectively, these findings suggest that the majority of DNA methylation changes during both development and maturation of the SE occur in distal regulatory regions, for example in enhancers.

TFBS motif analysis of DMRs, performed in order to examine the regulatory network (Fig. 3b; Supplementary Table S5), revealed the enrichment of a number of known inner ear regulators, including Six1^34^, Stat3^35^, and Wnt factors Tcf3 and Tcf4^36–38^. Another factor found in both LMRs and DMRs was Sox2, a key factor in the prosensory differentiation of hair cells^39^. Interestingly, the motif for Atoh1 is hypermethylated by P22 (MatTrans hyper-DMRs), in accordance with the down-regulation of Atoh1 after P5, and its early role in major cell type differentiation processes. The Bach2 motif was enriched only in MatTrans hypomethylated DMRs (P = 1 * 10^−5^), and added to its enrichment pattern in LMR-predicted TFBS (Fig. 2e).

In order to predict putative target genes for DMRs, we used known interactions from the 4DGenome database and predicted interactions from the PreSTIGE database^29^. We then leveraged the DMR motif analysis of TFs and putative target genes to construct DevTrans and MatTrans *in silico* regulatory networks and investigate any associated biological processes (Fig. 3c–e; Supplementary Tables S6 and S7). To reduce the complexity of regulatory networks, we chose to focus on a few specific transcription factors that are well known for their role in the inner ear and to use these as the basis for the creation of centralized networks. For DevTrans, we centered the network around Stat3 (Fig. 3c), a factor known to play a role in hair cell lineage determination and possibly regenerative capacity^35,40^. One GO term that stood out was, ‘signaling pathways regulating pluripotency of stem cells’ (KEGG:04550 DevTrans adj P = 0.00149, MatTrans adj P = 0.0039), which illustrates that pluripotency factors such as Stat3, Sox2, Tcf3, Smad4, and Wnts are also key for regulation in the SE. Other connected processes of interest were ‘HIF-1 signaling^41,42^, previously implicated in noise protection and ‘Lipid homeostasis’, which while largely unexplored in the auditory system, has been associated with neurodegenerative processes. Furthermore, there was an exclusive Atoh1-Stat3 interaction around the ‘Notch signaling pathway’ in the Stat3 centralized network, which may indicate a possible role of DNA methylation in the Notch signaling response in hair cells.

The network centered around Sox2 (Fig. 3d), a key TF that drives cochlear neurogenesis and other types of supporting cells^43,44^, revealed possible interactions involved in regulating the sensory perception of sound (GO:0007605) during the SE maturation phase. Our analysis also suggests an interaction of Sox2 with various cell-to-cell adhesion-related genes (i.e. *Cdh1*) and stereocilia formation-related genes (i.e. *Pcdh15* and *Myo3a*)^45,46^ under the umbrella of sensory perception of mechanical stimulus (GO: 0050954). Interestingly, ‘response to lipid’ (GO:0033993) was enriched for Sox2 target genes and revealed the involvement of CEBPß and its target genes, an observation in accordance with the TFBS analysis of LMRs.

### Time point-specific DNA methylation changes

**Figure 4:**
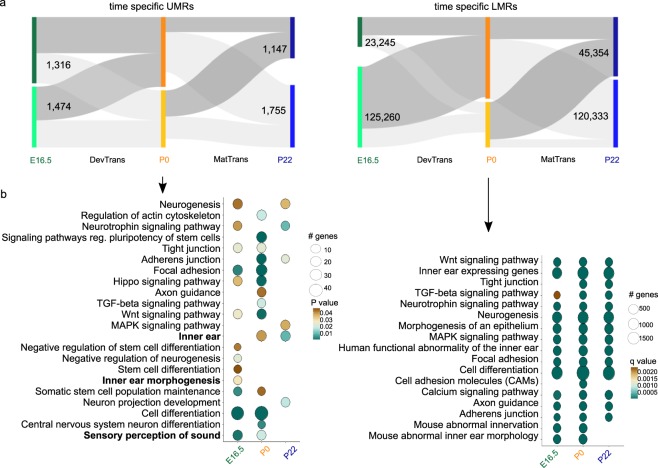
Analysis of time point-specific unmethylated region (UMR) and low-methylated region (LMR) putative regulatory regions. (**a**) Time point-specific UMRs (5,692) and LMRs (314,192) and their dynamics through the time points, represented as Sankey plots; regions defined at a specific time point as UMR/LMR (dark grey) and regions not defined as UMR/LMR (light grey). (**b**) GO term enrichment analysis of time point-specific UMR (top) and time point-specific LMR (bottom) associated genes; for UMR analysis, Circle fill is according to P value (<0.05) and for time point-specific LMR according to q value (FDR < 0.05). Circle diameter is proportional to the number of genes associated with each term.

Changes in DNA methylation are subject to thresholds that can be arbitrary. Our requirement for a DMR to have 30% change in overall methylation may have underestimated the dynamics of LMR methylation, and even to a certain extent that of UMRs (Supplementary Fig. S3a,b). Specific TFBS might be altered by much smaller changes that are not reflected in the average methylation change of DMRs, we therefore also determined 5692 UMRs and 314,192 LMRs that were identified at a single time point as additional sites of interest. These are referred to as time point-specific UMRs and LMRs (Fig. 4a).

We associated time point-specific UMRs to nearby genes and performed GO term enrichment analysis (Fig. 4a,b; Supplementary Fig. S3a and Table S8). The results revealed a number of enriched time point-specific terms, such as ‘sensory perception of sound’ (E16.5 and P0) for UMRs at *Ush1c*, *Coch*, and *Lrig2*. Other examples of time point-specific terms are ‘inner ear morphogenesis’ (E16.5), and ‘inner ear’ (P0 and P22). Neuronal development-related terms were enriched, as well as the formation of neurons (‘neurogenesis’), and the connections between neurons (‘axon guidance’). An examination of pathways known to be relevant to the SE, such as Wnt, suggested an association between the promoter methylation status and pathway regulation (e.g. *Wnt9a*). Other pathways found to be enriched included neurotrophins and MAPK signaling pathways.

When examining time point-specific LMR putative target genes we noted enrichment of SE-related terms such as ‘inner ear’ expressed genes (e.g. *Atoh1*, *Lrig2*, etc.). Interestingly, analysis of the mouse SE methylome resulted in enrichment of human phenotype terms such as ‘functional abnormality of the inner ear’, ‘abnormal inner ear morphology,’ and ‘abnormal innervation’, involving genes such as *Gjb2*, *Gjb6* and *Myo6* (Supplementary Table S9). Another feature we chose to highlight was the enrichment of immune-related terms for both time point-specific UMR- and LMR-associated genes. One example is TGF-ß signaling, which exhibits LMRs associated with BMP and SMAD gene families (Fig. 4b; Supplementary Tables S8 and S9).

### Gene expression and DNA methylation

**Figure 5:**
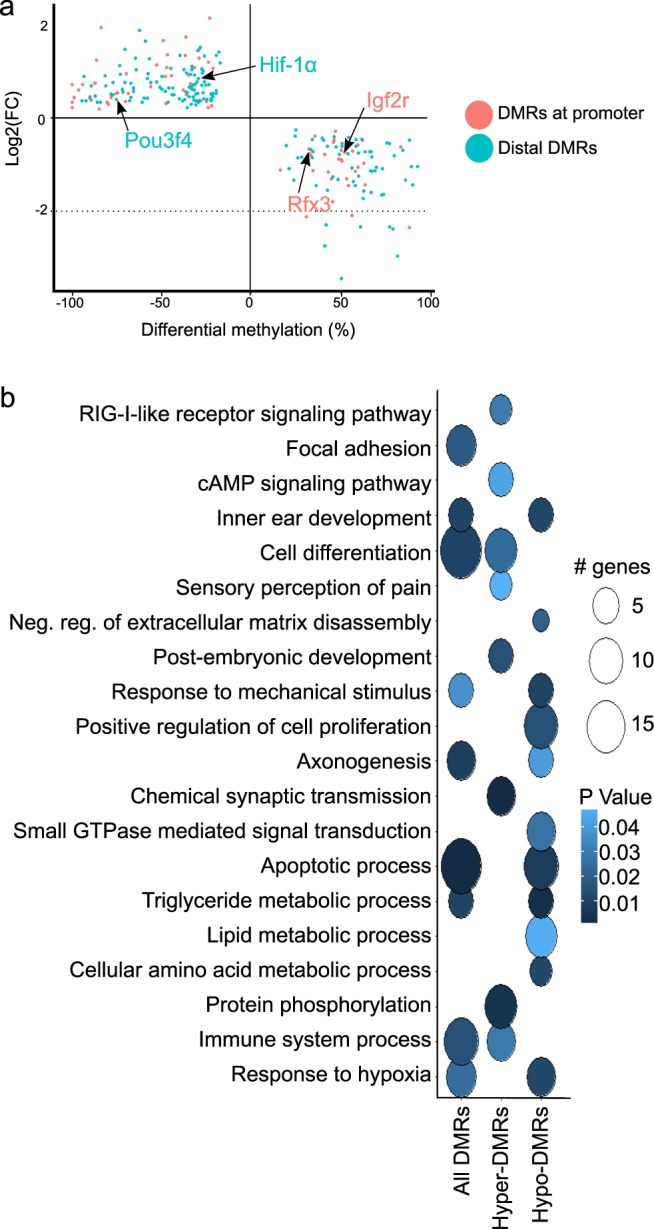
Gene expression and DNA methylation correlation for DevTrans. (**a**) Scatter plot of the differential methylation (P0/E16.5, percentage) at promoter and distal DMRs plotted against log2 of the fold change for the RNA-seq expression data (P0/E16.5, RPKM) of the putative target gene. Only genes whose expression is anti-correlated with the methylation level at associated DMR are shown, that is, expression increases (FC is positive) and methylation decreases (difference is negative) or vice versa. (**b**) GO term enrichment analysis of genes shown in **a**. Circle fill is according to P value (<0.05) and diameter is proportional to the number of genes associated with each term.

In order to investigate how DNA methylation changes may affect gene expression, we correlated transcriptomic data previously generated for E16.5 and P0^8–10^ with DMRs for DevTrans, focusing on DMRs located at promoters or distal to TSS (>5 kb) with an expected anti-correlation between gene expression and DNA correlated with expression of their putative target genes (Fig. 5a) including a key inner ear transcription factor, *Pou3f4*, which is required for hair cell development^47^. Upstream enhancers of *Pou3f4* have been previously implicated in causing X-linked deafness type 3 (DFN3)^48^. Our results showed that *Pou3f4* expression was up-regulated during DevTrans (0.455-fold increase, adjusted P-value = 0.00013), while its associated distal DMR was hypomethylated (%methylation difference = −67.15%, P < 0.0001). Other genes identified included *Hif-1α*, already identified in the DMR TFBS motif analysis (Fig. 3c,d), and members of the ‘Hif-1α signaling pathway’ highlighted in our DMR-based network analysis (Fig. 3c,d). GO analysis on the 255 anti-correlated hyper- and hypo-DMR genes (Fig. 5b; Supplementary Table S10) highlighted organ-relevant terms such as ‘inner ear development’ (hyper-DMR, P-value = 0.03) and ‘focal adhesion’ (combined, P-value = 0.045). Interestingly, genes with a negative correlation between DMR and gene expression dynamics, specifically hyper-DMRs (found either in promoter or distal to TSSs) were found to be enriched for the ‘immune system process’ (P-value = 0.03, respectively), while the hypo-DMRs were enriched for ‘response to mechanical stimulus’ (P-value = 0.001). In accordance with our observations of Hif-1α (described above), we noted ‘response to hypoxia’ (P-value = 0.012) GO term enriched among hypo-DMRs anti correlated to gene expression. Our results suggest that although derived from a heterogeneous cell population, focusing on genes whose expression is inversely correlated with DNA methylation during development, can lead to the identification of tissue relevant genes and pathways.

### Mouse inner ear SE LMRs are informative of regulation of human deafness

**Figure 6:**
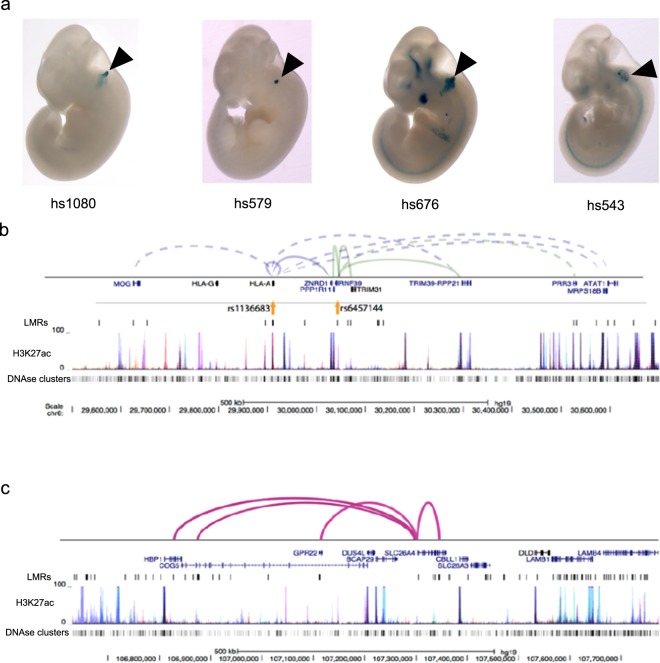
Inner ear sensory epithelium (SE) low-methylated regions (LMRs) are informative about human deafness. (**a**) Examples of experimentally validated mouse non-coding fragments with otic (ear) enhancer (black arrowhead) activity as assessed in transgenic mice from the VISTA Enhancer Browser for which we found overlap with lifted over LMRs at one of the three time points. (**b**) Browser shot of hearing-related variants at mouse-to-human LMRs and their target gene interactions on chromosome 6p21. Known interactions are shown as solid lines, predicted interactions as dashed lines. (**c**) Browser shot of mouse-to-human LMRs and known interactions around the Pendred syndrome gene *SLC26A4*. Layered H3K27ac from seven cell types and DHS data are shown in (**b**) and (**c**) to illustrate how SE LMRs can assist in the identification of *cis*-regulatory elements of interest.

The annotation of *cis*-regulatory elements in the human genome has increased our understanding of disease-associated variants by revealing their location in regulatory elements such as enhancers^49–52^. Here, we investigated how mouse inner ear SE LMRs could expand our understanding of human deafness, by using them to annotate putative regulatory elements in the human genome. We converted our mouse genome (mm10, *Mus musculus* genome assembly GRCm38, Genome Reference Consortium) LMR coordinates to human genome (hg19, *Homo sapiens* genome assembly GRCh37, Genome Reference Consortium) coordinates using the UCSC LiftOver tool. This approach allowed us to recover 56,439 LMRs at E16.5, 83,299 at P0, and 67,545 at P22. An average of 84% converted mouse-to-human LMRs were both hypersensitive (DHS) and marked by H3K4me1 in at least one human cell or tissue type^25^, supporting the assumption that these elements act as enhancers in the human genome. As validation that our mouse-to-human converted LMRs function as enhancers, we found an overlap of functionally validated enhancers from the VISTA Enhancer Browser repository, recovering 9 out of 19 human otic enhancers (Fig. 6a).

Next, we examined whether hearing-related variants from genome wide association studies (GWAS) and their proxy SNPs in linkage disequilibrium (LD) overlapped our mouse-to-human LMRs. Although most hearing-related studies have a limited number of associated variants, we succeeded in identifying 48 variants associated with hearing impairment, age-related hearing impairment, ear morphology and ear protrusion^53^ in LMRs from all time points (Supplementary Table S11). All but four of the LMRs are marked by H3K4me1 in at least one human cell or tissue type, suggesting that they are enhancer elements. By employing the same type of known and predicted 3D interactions described above, we were able to assign target genes to 19 of the variants (Supplementary Table S11). Many of these interacting variants were clustered on chromosome 6, primarily in 6p21 and 6p22 (Fig. 6b). Chromosome 6 is a hotspot of non-syndromic deafness genes, harboring seven autosomal recessive genes or loci (four on 6p21), and six autosomal dominant genes or loci (three on 6p21). Two autosomal dominant loci, DFNA21 (6p21) and DFNA31 (6p21.3) remain undetermined^54,55^.

We also examined the regions associated with deafness loci with unknown genes described in the Hereditary Hearing Loss Homepage (http://hereditaryhearingloss.org/) and performed a related reciprocal analysis. Chromosomal regions derived from microsatellite markers or coordinates of cytogenetic bands were located in the relevant manuscripts, identified in hg19, and converted to the homologous mouse interval (mm10). We identified SE LMRs in all of these regions, suggesting they may be associated with deafness in humans. These mouse-to-human maps provide a valuable resource for annotating putative regulatory elements relevant to the genetics of human deafness.

A number of affected individuals with hereditary hearing impairment do not harbor any known causative coding variants. For example, although almost half the cases of Pendred syndrome, a disorder that includes hearing impairment due to inner ear malformations in the cochlea and enlarged vestibular aqueduct (EVA), are caused by mutations in *SLC26A4*^56^, the cause of the remaining cases is largely unknown. It is therefore feasible that some manifestations of hearing impairment are caused by distal regulatory variants and that mapping mouse LMRs from the inner ear SE to human coordinates could direct the search for human *cis*-regulatory elements of known deafness genes. We have illustrated such an example for *SLC26A4*, where several mouse-to-human LMRs are near the gene, and at least four have known interactions (Fig. 6c).

### The human GJB2-GJB6 locus harbors a putative enhancer modulating GJB6 expression

**Figure 7:**
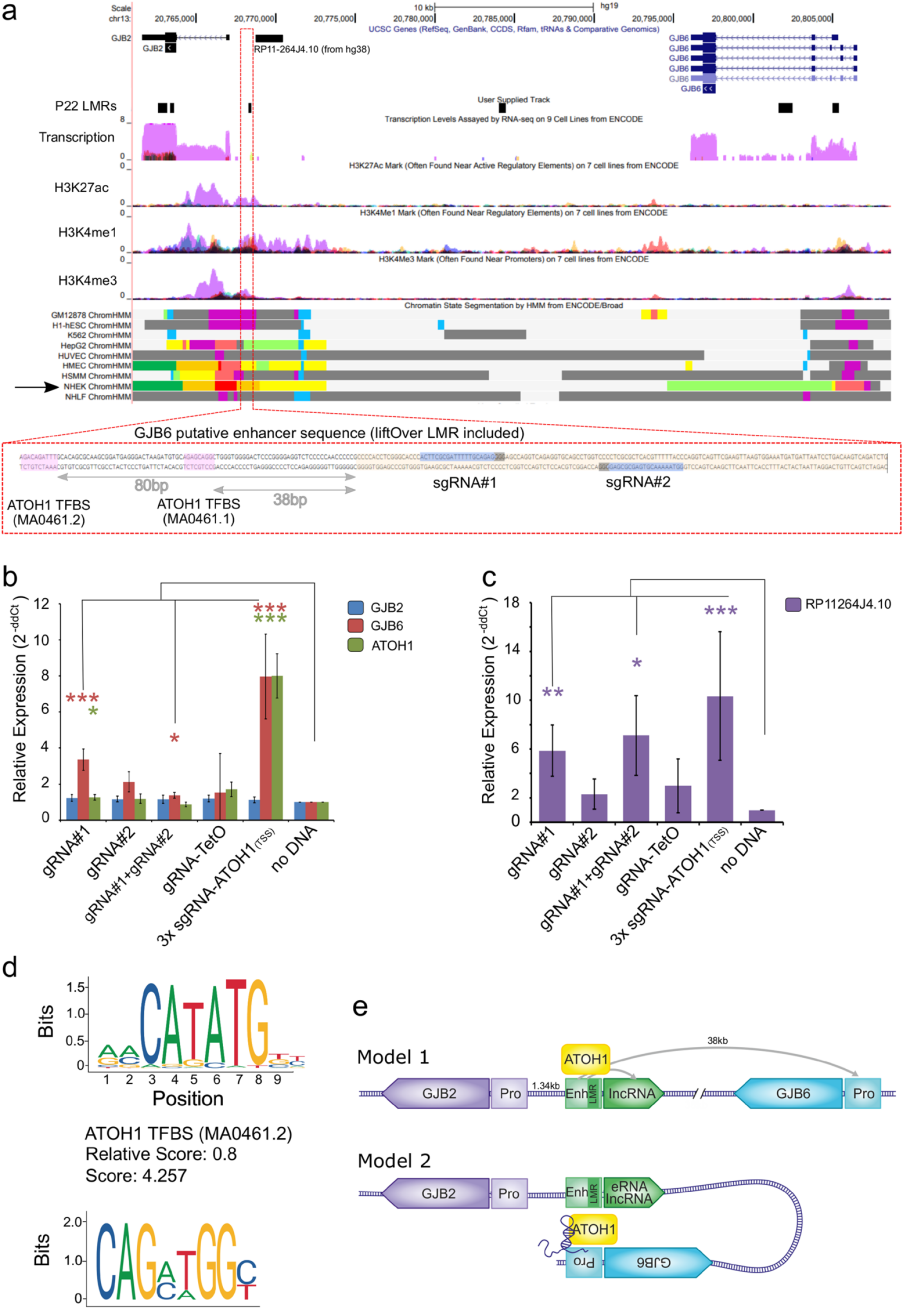
CRISPR-on modulation of *GJB6* and non-coding lncRNA *RP11-264J4.10* expression via a putative enhancer. (**a**) UCSC browser snapshot presenting the candidate enhancer lifted over from mouse (mm10) to human (hg19) (track: “P22 lifted LMRs”, black box indicators); additional information derived from ENCODE regulation hub is presented to support the characterization of the candidate sequence as an enhancer (track: ‘transcription’ - purple signal = NHEK cell line, track: ‘NHEK ChromHMM’ – orange indicating ‘Active enhancer’). Below, zoomed view of the candidate sequence, with the location of the lifted-over LMR, gRNA targets and ATOH1 TFBS indicated. (**b**, **c)** Expression of *GJB2*, *GJB6* and *ATOH1* (**b**) and *RP11-264J4.10* ncRNA (**c**) measured by qRT-PCR (normalized to *GAPDH*, compared to ‘no DNA’, n = 4–5, ***p < 0.01, *p < 0.05). (**d**) ATOH1 transcription factor binding motif found at the candidate enhancer, score is calculated by JASPAR. (**e**) Suggested mechanism of *GJB6* regulation by the putative enhancer, shown in two models. Model 1 depicts a physical distal interaction between the *GJB2* proximal putative enhancer (yellow box) and the *GJB6* promoter (light orange box) mediated by ATOH1 and/or the lncRNA (purple). Model 2 demonstrates the folding of the chromatin, linking the putative enhancer with the *GJB6* promoter, mediated by ATOH1 and/or the lncRNA.

In order to further validate that mouse-to-human LMRs can operate as gene enhancers, we utilized the CRISPR-on system^57^ and focused on the *GJB2* proximal regulatory region 1.34 kbp upstream of the *GJB2* TSS (Fig. 7a). This region is located between *GJB2* and *GJB6*, two prominent deafness genes, where *GJB2* is associated with approximately 30–50% of cases of deafness^58^. According to ENCODE data, this LMR is marked as an ‘active enhancer’ by ChromHMM segmentation in a *GJB2* and *GJB6* expressing cell line, NHEK (Normal Human Epidermal Keratinocytes) (Fig. 7a), confirming our prediction of this region as a putative enhancer in the inner ear SE. Two guide RNAs (gRNA) designed to target the *GJB2* proximal enhancer (Fig. 7a) were transfected into HEK293 T (human embryonic kidney) cells that express neither *GJB2* nor *GJB6*. Neither of the guides, individually or in combination, could modulate *GJB2* expression (Fig. 7b), when compared to the control (‘no sgRNA’) sample, despite the proximity to the *GJB2* promoter region (1.34 kbp between the LMR and *GJB2* TSS). Surprisingly, use of gRNA#1 alone resulted in a 3.36-fold increase in *GJB6* expression levels (P = 0.0043, n = 4), although gRNA#2 had no significant (P > 0.05) effect. While a combination of both gRNAs significantly increased *GJB6* expression levels, the effect was lower than gRNA#1 alone (1.37-fold, p = 0.035, n = 4). A gRNA targeting TetO was used as a negative control. Examination of the putative enhancer and the lifted-over LMR at its core, using the JASPAR core TF motif search tool^59^, detected two ATOH1 binding motifs, 38 bp (GACAGATTTG, JASPARScore = 4.257) and 80 bp (GAGCAGGC, JASPARscore = 3.311) upstream of the LMR and still within the putative enhancer (Fig. 7a,d). Based on this finding, we used the same CRISPR-on system to modulate *ATOH1* expression, which also served as an internal positive control, indicating that the CRISPR-on system transfection had worked properly. Enhancing *ATOH1* expression via CRISPR-on using three closely spaced sgRNAs targeting the *ATOH1* TSS also enhanced *GJB6* expression, but had no effect on *GJB2* expression (Fig. 7b). Targeting the putative enhancer with gRNA#1 also increased *ATOH1* expression by 1.27-fold (P = 0.047, n = 4). We further expanded our search to our RNA-seq data, where we identified a lncRNA, *XLOC_012867*, downstream to this LMR^10^. In the human hg38 genome, we detected a ncRNA transcript, indicated as *RP11–264J4.10* (GenBank AF091526.1), which was previously annotated as a *GJB2* ‘upstream regulatory region’ based on its proximity to the *GJB2* gene^60^. *R11-264J4.10* fits the criteria of a lncRNA at > 200 bp long (1,594 bp) and no open reading frame. Using the CRISPR-on system against the enhancer/LMR resulted in an expression modulation pattern similar to the one observed for *GJB6* (Fig. 7c), where gRNA#1 increased the lncRNA expression by 5.87-fold (P = 0.024, n = 5) and activation of *ATOH1* expression increased the lncRNA by 10.3-fold (P = 0.042, n = 5) (Fig. 7c). This might indicate that the enhancer interacts with multiple genes and is regulated through ATOH1 binding. Our data suggests that by lifting over mouse LMR putative enhancers to the human genome, we have annotated a relevant human enhancer region proximal to the *GJB2* gene and regulating the distal *GJB6* gene and a previously annotated ncRNA. Our observations also suggest an interaction between ATOH1 expression and *GJB6* regulation. While expression of these two genes is mutually exclusive in the differentiated SE, a possible intermediate stage could call for this regulatory interaction as early as the creation of the sensory primordium^61^.

## Discussion

The link between DNA methylation, its dynamics, and the development and differentiation of tissue, cellular lineages, and cellular functionality has been well established. Although DNA methylation has been studied extensively in both mouse and human in many tissues, the inner ear SE has been lacking any high-resolution characterization of this crucial epigenetic mark. Our work provides the first DNA methylome map in the mammalian inner ear SE, covering two major transitions, the developmental embryonic stage between E16.5 and newborn (P0) mice, and across the maturation transition period soon after the onset of hearing. The DNA methylation data generated should serve as a relevant resource for the auditory research field and provide information about inner ear development, deafness, and hearing impairment genetics, from the realm of coding and non-coding genes, into epigenetics and regulatory regions of the genome.

In this context, there have been previous reports that Notch factors are robustly activated in cells surrounding Atoh1-expressing cells and play a crucial role in supporting cell lineage determination and maturation^62^. Another example is the association of Tcf3 with lipid metabolism and regulation of stem cell states detected in our networks focused on supporting cell or hair cell TFs respectively. Tcf3 is known to play a role in inner ear SE development^63,64^, and to repress Wnt-ß-catenin signaling in neural precursor cells^37^ in order to maintain the undifferentiated state. Taken together, we can use our results to postulate an interplay between lipid metabolism and pivotal signaling pathways in SE development, and the potential to harness external lipid delivery to modulate signaling pathways. HIF-1α was another factor identified in our study as regulated by changes in the DNA methylation of regulatory elements. While very little is known about the role of HIF-1α in the mouse inner ear, there is evidence that exposure to noise induces HIF-1α expression, and that up-regulation of HIF-1 signaling protects the mouse from noise induced hearing loss^41,42^. The Stat3-centric DMR network, therefore, suggests that HIF-1 signaling plays a role in tissue maturation. Collectively, these networks, which are anchored on DNA methylation dynamics, reveal epigenetic and regulatory insight on the development and maturation of the inner ear SE.

Our survey of regulatory regions in the mouse SE, based on the methylome analysis, will be an important source of information to prioritize non-coding variants identified in human families as whole genome sequencing (WGS) is increasingly becoming the method of choice for identifying pathogenic variants. As evidence that LMRs in the SE function as *cis*-regulatory elements, we have demonstrated that they harbor chromatin marks indicative of enhancers, such as DHS and H3K4me1 in other cell types. Moreover, the LMRs were shown to function as otic enhancers in transgenic assays presented in the VISTA Enhancer Browser, and we provide CRISPR-activation validation for one such candidate LMR, which was found in the *GJB2-GJB6* locus. Using the CRISPR-on system targeting this putative regulatory element, we showed expression modulation of both *GJB6* and a ncRNA, with as yet undefined function, but not the proximal *GJB2* gene itself. Both *GJB2* and *GJB6* encode key structural proteins in the inner ear SE; therefore, their expression is likely to be tightly governed by key inner ear differentiation and maturation TFs. Taken together with known lncRNA acting as co-factors in modulating transcription, we can hypothesize two plausible models of operation connecting all factors at hand – *GJB2*, *GJB6*, ATOH1, lncRNA and the LMR/enhancer (Fig. 7e). In the first model, a classic enhancer model is at play, where the LMR/enhancer is bound by ATOH1 and chromatin looping facilitates interaction with the promoters for the lncRNA and *GJB6*. This looping excludes regulation of the nearby *GJB2 gene*. A second plausible model is based on chromatin looping, directly linking the putative enhancer with the *GJB6* promoter, and involves a role for the lncRNA. The enhancer-promoter interaction could be stabilized by the lncRNA, creating a small “niche”, excluding the *GJB2* promoter and gene from regulation over *GJB6*. Given that we have numerous other potential candidates to evaluate using this CRISPR-on validation approach, this methodology can also be employed in a high-throughput screen to validate converted mouse-to-human regulatory regions.

In conclusion, this study describes the first DNA methylome map of the mammalian inner ear SE and indicates that a genome-wide perspective of DNA methylation may provide valuable information about the processes of inner ear lineage formation, tissue morphogenesis, and gain of auditory function. Although these results represent the development of the SE tissue as a whole, future studies could concentrate on the DNA methylation and epigenetic regulation of specific pathways or cellular populations. Finally, increasing our knowledge of the role of a global epigenetic regulatory process, as well as the dynamics of regulatory elements, may advance regenerative research of the inner ear sensory organ by directing the manipulation of gene expression, or repurposing currently available epigenetic therapeutics in order to treat or prevent the onset of hearing impairment.

## Materials and Methods

### Animals and SE preparation

All procedures involving mice met the guidelines described in the National Institutes of Health Guide for the Care and Use of Laboratory Animals and were approved by the Animal Care and Use Committees of Tel Aviv University (M-13-114, M-13-115 and 01-16-100). C57BL/6J mice were purchased from Envigo, Jerusalem, Israel. The ages of the mice used were E16.5, P0 and P22. Two replicate groups of 6–8 mice each was used for each age group. Pregnant dams or postnatal mice were sacrificed by CO_2_ suffocation, followed by immediate decapitation; only decapitation was employed when dissecting newborns. The skin at the top of the head was removed and the dorsal part of the skull was opened along the midline. The brain tissue was removed and the auditory nerve was pulled out and cut. The inner ear, containing both the cochlea and the vestibule, was removed from the temporal bone, and placed in phosphate buffered saline (PBS). The cochlea was further dissected; the otic capsule, the spiral ligament and the stria vascularis were removed to expose the SE. After this, the SE was separated, base to apex, from the spongy modulus bone. Both SE from each group were pooled together to generate two distinct biological replicates for each age group.

### Whole genome bisulfite sequencing

We performed WGBS on SE using the MethylC-Seq protocol^65^, with minor modifications. Genomic DNA was purified from replicate samples of pooled SE from both ears of 6–8 mice, using the QIAamp DNA Micro gDNA Kit (Qiagen). Each sample was spiked with 0.5% of total gDNA mass with Lambda unmethylated DNA to serve as an internal control for bisulfite (BS) conversion rates. gDNA samples were fragmented using the COVARIS S220 with Snap-Cap microTUBE with AFA fiber 6 × 16mm (Covaris). Illumina-compatible NGS libraries were produced using the NEBNext® Ultra DNA Library Prep Kit for Illumina® (NEB) and Multiplex Oligos for Illumina® (Methylated Adaptor, Index Primers Set 1) (NEB). Adaptors were ligated and the library DNA was cleaned and size selected using Agencourt AMPure XP (Beckman Coulter) magnetic beads. BS conversion was performed using Methylcode Bisulfite Conversion Kit (Applied Biosystems). The library DNA was PCR amplified through 4–6 cycles using KAPA HiFi Uracil + polymerase with indexed and universal Illumina compatible oligos. The library PCR was cleaned and size selected on AMPure XP beads. Library concentration was validated using the Qubit dsDNA High Sensitivity platform, and the size distribution was assessed with the DNA High Sensitivity Kit (Agilent). NGS was performed at BGI, China on an Illumina HiSeq 2500. Libraries were divided equally across lanes to minimize technical bias during sequencing.

### Methylation data analysis

WGBS reads were aligned to the mouse reference genome (mm10 assembly) using Bismark (version v0.14.0)^66^ with default options and duplicates were removed using SAMtools (0.1.18)^67^. The library fold coverage was computed by dividing the number of uniquely aligned reads by the size of the genome. The mapping efficiency was computed by Bismark using the number of unique paired end alignments against the total paired end reads. The bisulfite conversion rates were computed by Bismark after mapping each sample with the lambda phage genome. Differential methylated regions (DMRs) were called using methylPipe (version 1.4.5)^68^. A > 30% methylation difference and a non-parametric Wilcoxon test P-value < 0.05, corrected for multiple testing, were applied as cutoff. Unmethylated and low methylated regions (UMRs and LMRs) were identified using the R package MethylSeekR^23^, using the following criteria: (i) FDR < 5% for regions, (ii) average DNA methylation < 10% (UMRs) or < 50% (LMRs), (iii) mCG/CG and > 5 CGs per region. Regions with any significant overlap between UMRs and LMRs were removed from the datasets to prevent ambiguity.

### ENCODE data

H3K4me1, DHS, and CTCF binding site data peak calls from various cell types were downloaded from the ENCODE consortium^25^ and overlap analysis was performed using the BEDTools suite^69^.

### Prediction of target genes for regulatory regions

To predict the potential regulatory interactions of LMRs, UMRs and DMRs with their target genes, known interactions were obtained from the 4DGenome database (https://4dgenome.research.chop.edu/) and predicted interactions from the PreSTIGE database^70^. LMRs, UMRs and DMRs were cross-referenced with the interaction data to predict the target genes. The known human deafness genes were obtained from^30^.

### TFs analysis

TF motif enrichment analysis was performed using the HOMER Motif Analysis software (http://homer.ucsd.edu/homer/motif/)^71^. TF motifs with P-value < 1e-10 and TFs with expression, RPKM > 1 were selected from available inner SE RNA-seq datasets^8–10^. TF DNA binding preferences were predicted using JASPAR, http://jaspar.genereg.net/ ^59^.

### Gene regulatory networks construction

Two gene regulatory networks were constructed for each transition. TFBS motif enrichment analyses of hyper- and hypo-DMRs from the two transitions (DevTrans and MatTrans) were used to identify enriched TF motifs (P-value < 0.01). We then compared the identified DMRs with enhancer-gene interaction data from the 4DGenome and PreSTIGE databases and assigned the TFs, according to the enriched motif present at DMRs, to their interacting genes. The results were screened for gene expression (differential expression, DEGs) and for anti-correlation, i.e. hyper-DMRs should interact with down-regulated genes and hypo-DMRs with up-regulated genes and for TFs with RPKM > 1. Network visualizations were created using the Cytoscape software tool^72^.

### Gene ontology analysis

TSS proximal regulatory features were analyzed for GO terms and KEGG pathway using DAVID (https://david.ncifcrf.gov/)^73^ with default parameters. Terms were ranked according to the P-value (EASE score), which is derived from a modified Fisher’s exact test. Associated genes were determined according to previously described genomic annotation of the TSS proximal genomic ranges datasets, derived from lists of official gene symbols or ENTERZ gene id numbers (for lists > 3000 genes long).

For TSS distal elements, analysis of GO term enrichment was executed using the HOMER Motif Analysis software (annotatePeaks.pl hg19 -go)^71^ for DMRs or GREAT (http://bejerano.stanford.edu/great)^74^ for putative regulatory elements characterized as time point-specific LMRs. Terms were ranked according to the Binomial test Q score (Binom FDR Q-Val < 0.05) and the Hypergeometric test Q score (Hyper FDR Q-Val < 0.05).

### Identification of proxy SNPs and LMR liftover

SNPs for available hearing-related impairment were downloaded from the NHGRI-EGI GWAS Catalog (https://www.ebi.ac.uk/gwas/). LD SNPs were determined using rAggr, max distance 500 kb (http://raggr.usc.edu). An R-squared threshold of 0.5 was used based on previous studies showing enrichment at distal *cis*-regulatory elements for SNPs from r2 1.0 to 0.5^75,76^. Mouse mm10 coordinate LMRs were converted to human hg19 coordinates using the UCSC LiftOver tool (https://genome.ucsc.edu/cgi-bin/hgLiftOver).

### CRISPR-on system

We used the pAC154-dual-dCas9VP160-sgExpression^57^, a gift from Rudolf Jaenisch (Addgene plasmid # 48240), and designed gRNAs using the CHOPCHOP web tool (http://chopchop.cbu.uib.no/)^77^ to select the top-ranking guides. Oligos suitable for cloning into the *Bbs*I site were ordered from IDT. The guide sequences were: gRNA#1 FWD 5′-caccGacttcgcgatttttgcagag-3′, gRNA#1 REV 5′-aaacctctgcaaaaatcgcgaagtC-3′, gRNA#2 FWD 5′-caccGgtaaaaacgtgagcgcgag-3′, gRNA#2 REV 5′-aaacctcgcgctcacgtttttacC-3′. Correct integration of guide RNAs into the plasmid was validated using Sanger sequencing with a sequencing primer from the U6 promoter upstream of the integration site, U6_SEQ. 5′CAAGGCTGTTAGAGAGATAA-3′. All cloning design was performed using the Benchling platform (www.benchling.com). Plasmids were harvested using NucleoSpin MidiPrep (MN#74010). Plasmids were transfected into 50% confluence cells seeded 18 hours earlier in 6-well plates. Transfection was performed using JetPEI reagent according to the manufacturer’s protocol (Polyplus#101). Cells were harvested for RNA 48 hours post-transfection using the ZYMO Direct-zol RNA Miniprep Kit (Zymo research# R2070). RNA was measured using the NanoDrop and 500 ng of RNA was taken from each sample to prepare cDNA using the qScript Reverse Transcription Kit (Quantabio 95047). All experiments were performed with two technical repeats of each sample and across 5 biological replicates.

### Real Time qPCR

The expression of mRNA was evaluated using the PerfeCTa SYBR® Green FastMix (QuantaBio) in the StepOneTM Real-Time PCR System (Applied Biosystems). Primers were designed using Primer3plus (http://primer3plus.com/cgi-bin/dev/primer3plus.cgi) with the default parameters to reach an amplicon of 80–150 bp. Oligos were ordered from IDT as a 100 µM stock. Primer sequences were: GJB2_FWD 5′AAAAGCCAGTTTAACGCATTGC'3, GJB2_REV 5′TTGTGTTGGGAAATGCTAGCG'3, GJB6_FWD 5′TGGCAAATTTGT GAACTGTCATG'3, GJB6_REV 5′TCAGTTGTTTGCAATGATTGGC'3, RP11–264J4.10_FWD 5′TGCTCATGAAGAGGCAAAGC'3, RP11-264J4.10_REV 5′TTAACAAGCCGACTCAGCAC'3. All samples were normalized to GAPDH endogenous expression with the following primers GAPDH 5′ GGAGCGAGATCCCTCCAAAAT'3 and GAPDH_REV 5′GGCTGTTGTCATACTTCTCATGG'3. The negative control was defined as the Non-Template Control (NTC). The relative expression level was measured using the 2-ddCt method. mRNA levels from un-transfected cells (termed “noDNA”) were defined as 1. The data in Fig. 7 are presented as the mean ± SE.

## Electronic supplementary material

Supplementary Information

Supplementary Table S1

Supplementary Table S2

Supplementary Table S3

Supplementary Table S4

Supplementary Table S5

Supplementary Table S6

Supplementary Table S7

Supplementary Table S8

Supplementary Table S9

Supplementary Table S10

Supplementary Table S11

## Data Availability

The datasets are available in the NCBI Short Read Archive (SRA); the accession number for the data generated from mouse inner ear SE is SRP111167. Access to SRA metadata can be found at: www.ncbi.nlm.nih.gov/sra, as well as within the article and additional files.
