# Supplementary material for: DNA methylation dynamics during embryonic development and postnatal maturation of the mouse auditory sensory epithelium
Source: Sci Rep. 2018 Nov 26;8:17348. doi: 10.1038/s41598-018-35587-x (PMC6255903; doi:10.1038/s41598-018-35587-x)
Supplement: Supplementary file 1 — Supplementary Information [file 41598_2018_35587_MOESM1_ESM.pdf]

Supplementary Information

DNA methylation dynamics during embryonic development and postnatal maturation of the mouse auditory sensory epithelium

Ofer Yizhar-Barnea[1†], Cristina Valensisi[2†], Naresh Doni Jayavelu[2], Kamal Kishore[3], Colin Andrus[2], Tal Koffler-Brill[1], Kathy Ushakov[1], Kobi Perl[1], Yael Noy[1], Yoni Bhonker[1], Mattia Pelizzola[3], R. David Hawkins[2*] & Karen B. Avraham[1*]

[1]Department of Human Molecular Genetics and Biochemistry, Sackler Faculty of Medicine and Sagol School of Neuroscience, Tel Aviv University, Tel Aviv 6997801, Israel. [2]Division of Medical Genetics, Department of Medicine, Department of Genome Sciences, Institute for Stem Cell and Regenerative Medicine, University of Washington School of Medicine, Seattle, WA 98195, USA. [3]Center for Genomic Science of IIT@SEMM, Fondazione Istituto Italiano di Tecnologia, Milano 20139, Italy. Correspondence and requests for materials should be addressed to K.B.A. and R.D.H. (emails: karena@tauex.tau.ac.il, rdhawk@uw.edu)

[†]Ofer Yizhar-Barnea and Cristina Valensisi contributed equally to this work

Contents

Figure S1. General features of inner ear SE methylomes. Related to Figure 1.
Figure S2. General features of DMR. Related to Figure 3.
Figure S3. Dynamics of UMRs and LMRs. Related to Figure 4.

Supplementary Tables are available as separate files

Table S1. UMR and LMR coordinates.
Table S2. Otic enhancers from the VISTA Enhancer Browser.
Table S3. UMR and LMR interacting genes.
Table S4. TFBS motif analysis for UMRs and LMRs.
Table S5. DMR coordinates and TFBS motif analysis.
Table S6. DMR TF motifs and interacting genes.
Table S7. GO enrichment analysis for regulatory networks.
Table S8. GO enrichment analysis of time point-specific UMRs.
Table S9. GO enrichment analysis of time point-specific LMRs.
Table S10. GO enrichment analysis of genes with an expected inverse correlation between DNA methylation and gene expression.
Table S11. Hearing-related variants from GWAS at lifted over to human LMRs and their putative target genes.

Abbreviations. SE: sensory epithelium; UMR: unmethylated region; LMR: low methylated region; DMR: differentially methylated region; TF: transcription factor; TFBS: transcription factor binding site; GO: gene ontology; DevTrans: developmental transition; MatTrans: maturation transition.

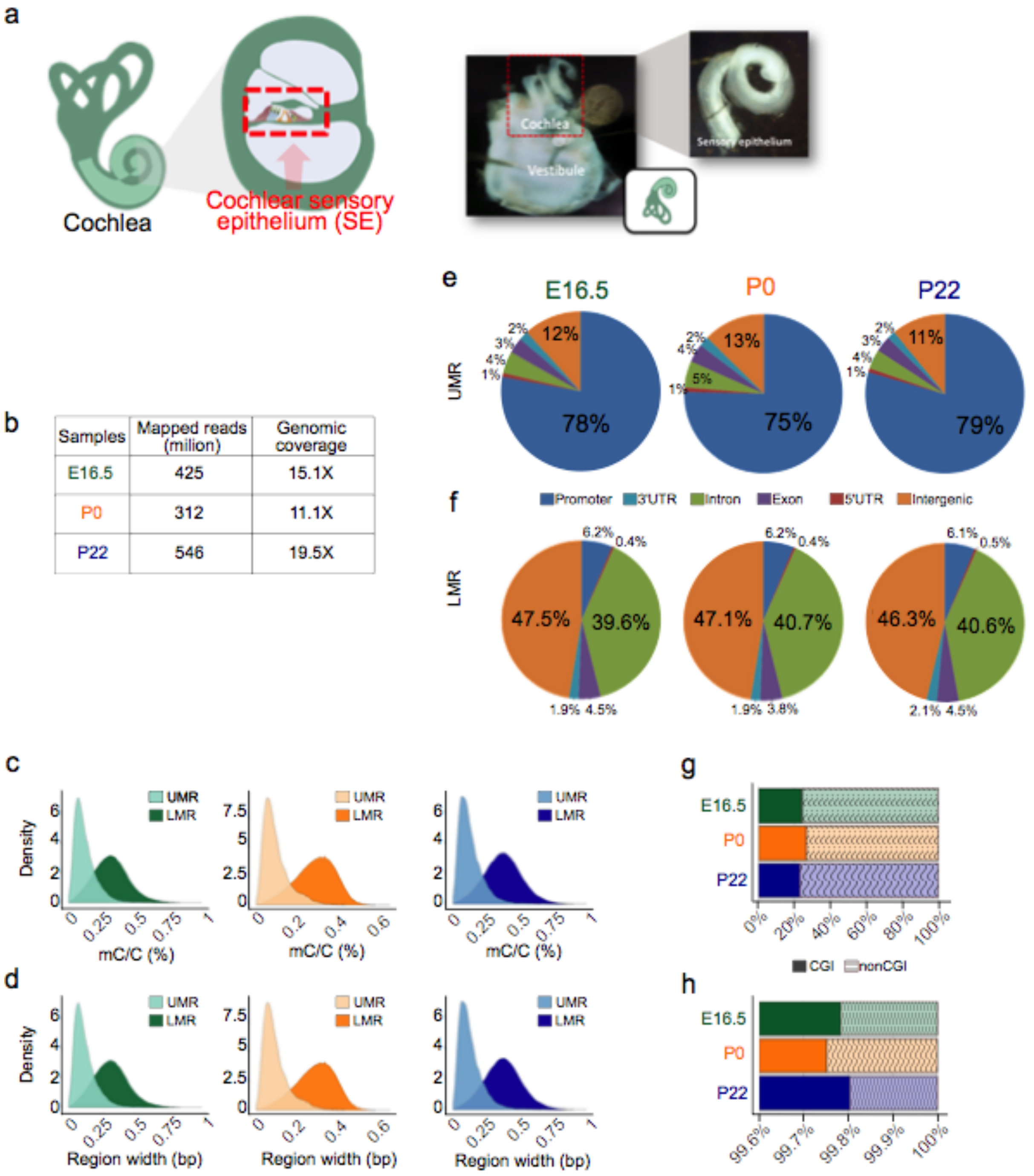

Supplementary Figure S1. General features of inner ear SE methylomes. (**a**) The SE (live image on the right) is dissected from the cochlea of the inner ear (left). Scale bar: 50 µm. (**b**) The table shows the number of mapped reads (in millions) and genomic coverage for the combined replicates at each time point (two replicates per time point). (**c, d**) Density plots showing the methylation levels for UMRs and LMRs, respectively, at each time point. (**e, f**) Pie charts showing the distribution of UMRs and LMRs, respectively, across genomic features. (**g, h**) Bar charts showing the overlap of UMRs and LMRs, respectively, with CpG islands (CGIs) - CGIs were obtained from the UCSC Genome Browser. Abbreviations. UMR: unmethylated regions; LMR: low methylated regions.

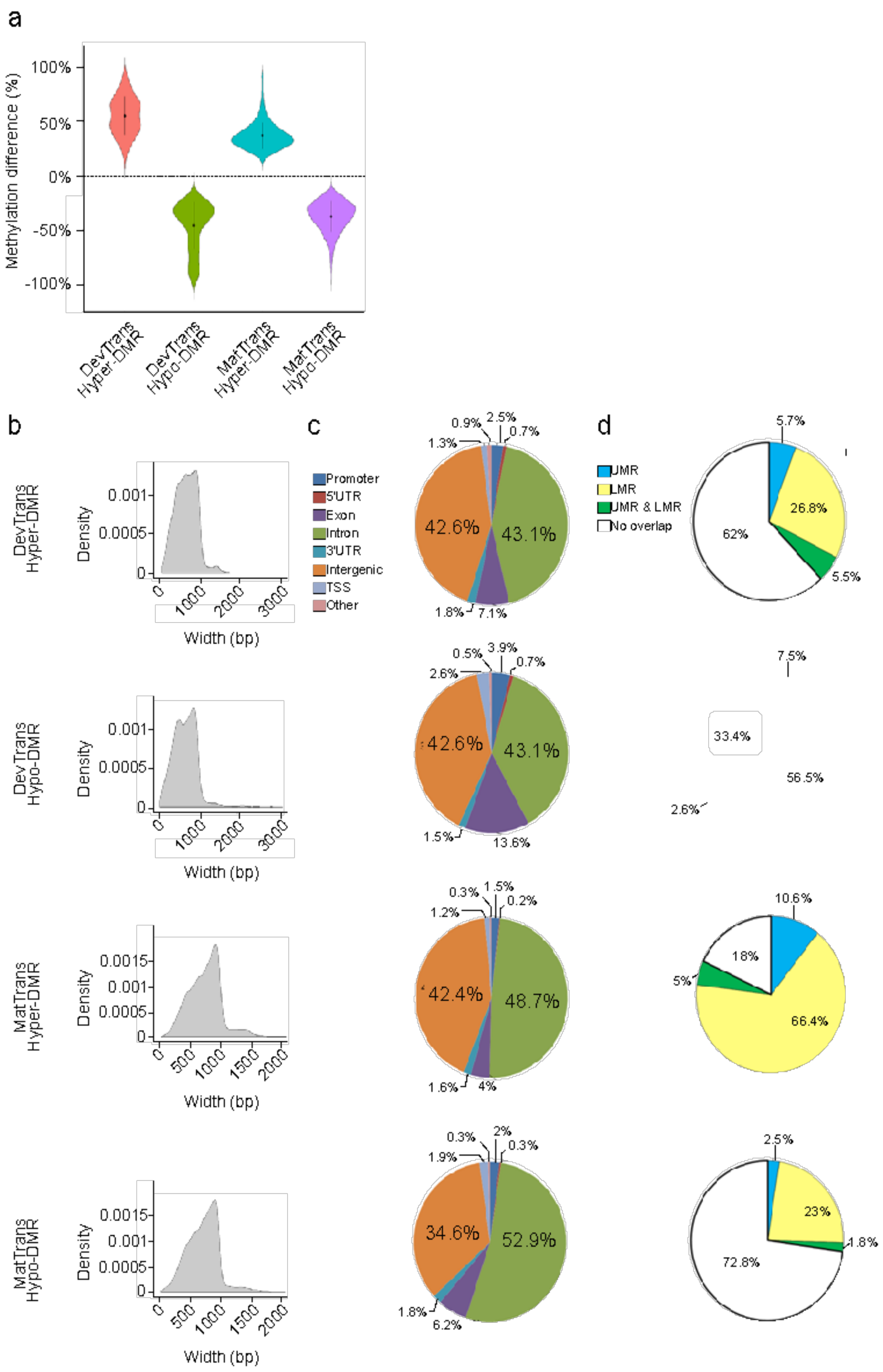

Supplementary Fig S2. DMR general characteristics. (**a**) Violin plots of the distribution of differential methylation for hypo- and hyper-DMRs for DevTrans and MatTrans transitions. The median (internal black dot) and standard error (internal black lines) are shown. (**b**) Density plots of DMR width (bp). (**c**) Distribution of DMRs genomic features. (**d**) Pie charts of DMRs that overlap with UMRs and LMRs. b-d) From top to bottom, DevTrans Hyper-DMRs, DevTrans Hypo-DMRs, MatTrans Hyper-DMRs, and MatTrans Hypo-DMRs are shown. Abbreviations. DMR: differentially methylated region; hypo- or hyper-DMR: DMR that undergoes a lost (hypo) or a gain (hyper) of methylation during the transition.

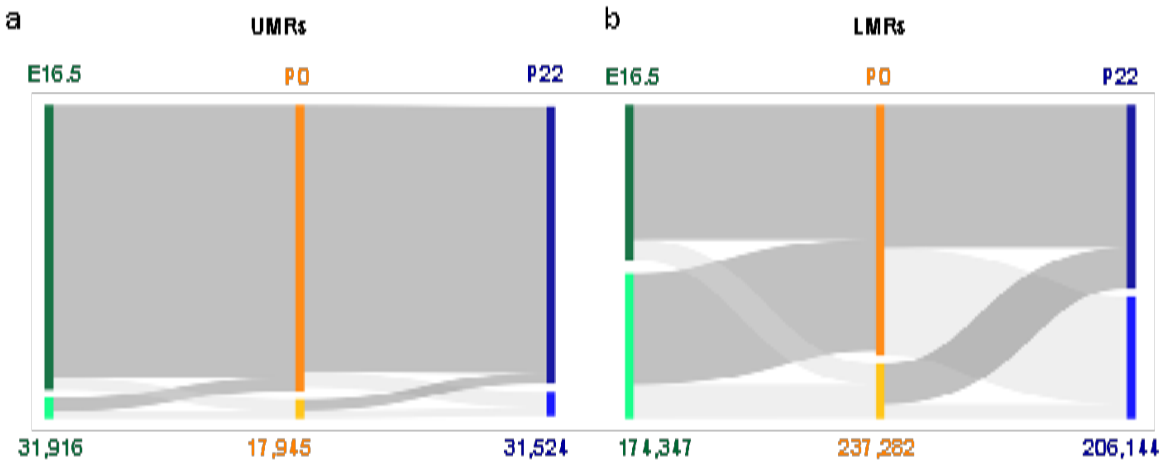

Supplementary Figure S3. UMR and LMR dynamics. (**a, b**) Sankey plot visualization of a 3-way intersection between the complete subset of UMRs at the three time points examined. All UMRs and LMRs are presented. Dark grey connections between nodes indicate regions that either maintained or acquired their UMR/LMR status during the transition. Light grey connections between nodes indicate regions that gained methylation during the transition and are no longer defined as UMRs (**a**), or LMRs (**b**) at the end of the transition. Abbreviations. UMR: unmethylated regions; LMR: low methylated regions.
